# The Effect of Pellet Diameter on the Growth Performance, Nutrient Digestibility, and Intestinal Health of Piglets During the Creep Feeding Stage

**DOI:** 10.3390/ani16081260

**Published:** 2026-04-20

**Authors:** Lingao Kong, Fangxing Ou, Shuang Dong, Nan Zhang, Yongxi Ma

**Affiliations:** State Key Laboratory of Animal Nutrition, Department of Animal Nutrition and Feed Science, College of Animal Science and Technology, China Agricultural University, Beijing 100193, China; kla011003@163.com (L.K.);

**Keywords:** creep feed, pellets, growth performance, intestinal morphology, intestinal microbiota

## Abstract

This experiment evaluated the effects of pellet diameter on growth performance and intestinal health of piglets during the creep feeding stage. A total of 144 7-day-old suckling piglets (body weight of 2.2 ± 0.3 kg) were randomly assigned to four groups and fed the same formula as meal feed and pellets of 2 mm, 4 mm, and 8 mm in diameter, respectively. Each treatment consisted of six replicates of six piglets. The trial was divided into two phases by weaning time: 7–21 days (breast milk + creep feed) and 21–35 days (creep feed only). After the feeding trial, piglets from the meal feed group and the 8 mm pellet group were selected for slaughter and sampling. The results showed that before weaning, average daily feed intake (ADFI) increased significantly with increasing pellet diameter (*p* < 0.001). Post-weaning, piglets fed 8 mm pellets presented significantly higher final body weight (FBW) and average daily gain (ADG) than those in the meal group (*p* < 0.05). Apparent nutrient digestibility (ATTD) in pellet groups was significantly higher than that in the meal feed group and rose with increasing pellet diameter (*p* < 0.001). The organ indices of the stomach and large intestine in the 8 mm group of piglets were significantly lower than those of the meal group. The jejunal villus height (VH) in the 8 mm group showed a trend toward an increase (*p* = 0.066), and the ileal crypt depth (CD) was significantly lower (*p* = 0.004), with significantly higher digestive enzyme activities in the jejunum and ileum (*p* < 0.05). In the 8 mm group, the relative abundances of Bacteroidetes in the jejunum and Actinobacteriota in the cecum and colon increased, while those of Pseudomonadota decreased; jejunal microbial relative richness increased significantly, while the ileal microbial operational taxonomic unit (OTU) richness decreased obviously. In conclusion, pellets improved the growth performance of creep feeding piglets. Compared with meal, 8 mm pellets can significantly enhance intestinal health level and nutrient digestion and absorption capacity by optimizing intestinal morphology, boosting digestive enzyme activities, and improving flora structure.

## Simple Summary

Compared with meal feed, the use of pellets as creep feed can better promote growth and nutrient digestibility, and increasing pellet diameter improved performance in a dose-dependent manner. Our experiment evaluated the effects of meal feed and 2 mm, 4 mm, and 8 mm pellets on the growth performance and intestinal health of piglets at the creep feeding stage. The results showed that the pellets were more suitable for piglets at the creep feeding stage than the meal feed, and the 8 mm pellets had the best comprehensive effect: they could significantly improve the feed intake and average daily gain and enhance nutrient digestibility and absorption capacity. At the same time, they can improve the intestinal morphology of piglets, increase intestinal digestive enzyme activities, and regulate the balance of flora. In conclusion, the use of large-diameter pellets in the early life of piglets resulted in improved growth performance.

## 1. Introduction

The creep feeding stage (7–35 days of age) is critical for piglets. Growth performance during this period influences overall production efficiency and economic outcomes. Piglets at this stage have immature digestive organs and immune systems [1,2]. Coupled with the environmental stressors, most piglets experience stress responses, which increase the difficulty of feeding and management practices [3]. For these reasons, ensuring the normal growth and development of piglets during the creep feeding stage is important for the swine industry. Among various challenges, weaning stress poses a major challenge to piglets during this phase. Studies have shown that post-weaning piglets exhibit reduced intestinal villus height (VH) [4] and increased crypt depth (CD) [5], which severely impair intestinal structure and function, leading to compromised digestive and absorptive capacity [6]. Therefore, only by addressing weaning stress can the healthy growth of piglets be ensured.

Feed is a key factor in pig production. Piglets grow and develop rapidly in the creeping stage and have high nutritional requirements, so feed quality often determines their production performance. High-quality creep feed should be formulated based on piglet requirements, such as imperfect digestive function and high nutritional requirements. It should fundamentally alleviate the problems associated with piglet weaning syndrome and possess good palatability [7]. To achieve this goal, feed pelleting technology has received increasing attention. Meal feed has several disadvantages, including increased dustiness, nutrient segregation, reduced palatability, and greater risk of respiratory irritation [8,9]. These issues can impair feed efficiency and increase feed wastage. In contrast, pelleting reduces dust, stabilizes nutrient uniformity, and improves physical structure. As a key parameter in the pelleting process, pellet diameter plays an important role in improving the growth performance of piglets during the creep feeding stage.

While creep feeding is widely recognized as critical for preparing suckling piglets for weaning, most studies have focused on nutritional composition, delivery method, and processing temperature, with limited attention to physical feed properties, especially pellet diameter [8,9]. Previous studies confirmed that larger pellet size increases ADFI and ADG of piglets during the creep feeding stage [10,11], but these findings remain descriptive and lack mechanistic evidence. Additionally, intestinal health is the core link determining nutrient digestibility. Available research has not clarified how pellet diameter regulates intestinal development, digestive enzyme activity, or microbial community structure in the pre-weaning period, and researchers have not established links among pellet physical characteristics, gut morphology, enzyme function, and microbiota. Therefore, knowledge regarding pellet size for suckling piglets remains insufficient.

Therefore, this study evaluated pellets with diameters of 2, 4, and 8 mm using piglets during the creep feeding period. It intended to examine the effects of pellet diameter on the growth performance and intestinal health indicators of piglets during the creep feeding stage, thereby offering theoretical and practical references for the improvement of creep feed. The pellet diameters were selected based on the previous literature investigating pellet size for suckling pigs [10,11]. These studies have evaluated a range of pellet diameters and suggested potential benefits of larger pellets, but no consensus exists regarding the optimal size. To systematically investigate the potential dose-dependent effects of particle diameter, we established a gradient grading series, which allowed us to identify performance across small, medium, and large pellet sizes relative to a meal feed control. We hypothesized that increasing the pellet diameter would improve the growth performance, nutrient digestibility, and intestinal health of piglets during the creep feeding stage, with the best effect observed at 8 mm.

## 2. Materials and Methods

The Animal Ethics Committee of China Agricultural University reviewed and approved all experimental protocols (Approval No. AW52404202-1-1), in compliance with national guidelines for animal welfare. Piglets were raised at the Feng Ning Swine Research Unit, a facility operated by China Agricultural University in collaboration with Chengdejiuyun Co., Ltd. (Chengde, China).

### 2.1. Experimental Designs

The first stage spanned from 7 to 21 days of age (weaning). In total, 144 crossbred piglets (Landrace × Yorkshire × Duroc) derived from 12 litters were randomly allocated to 4 treatments, with 3 litters and 12 piglets per litter in each treatment. The average initial body weight of piglets was 2.2 ± 0.3 kg, and no significant differences in average litter weight were detected among all groups. All sows were at 4–5 parities, with an average parity of 4.58. The second stage began post-weaning, following the separation of piglets from their sows. Within each original treatment, piglets from the 3 litters were redistributed into 6 new groups with similar average body weight, containing 6 piglets per group. Growth performance was systematically recorded during this stage.

All piglets were confirmed healthy by clinical observation (no diarrhea, fever, or abnormal behavior) before grouping, and sows were in good lactation status (no mastitis, consistent milk production during the experimental period) and were fed the same sow feed.

Dietary treatments were meal feed and pellets with diameters of 2, 4, and 8 mm. The three types of pellets were produced using ring dies with the same compression ratio. The trial commenced on day 7 of lactation and lasted until weaning at day 35, with a total period of 28 days. All diets were formulated using the same formula, and the diets (Table 1) were designed to satisfy the recommended requirements of suckling piglets [12]. Table 2 presents the physical quality of three types of pellets.

### 2.2. Feeding and Management

From the age of 7 days to 21 days, piglets and sows within each treatment were housed in the same pens; piglets received breast milk normally while feeding the creep feed, and only feed intake was measured during this period. All piglets were weaned at 21 days of age. From the age of 21 days to 35 days, piglets continued to be fed with the same creep feed as the first stage, and all growth performance indicators were measured. All piglets were provided with feed for ad libitum consumption throughout the trial period. Each replicate pen was fitted with a creep feed trough and a nipple drinker, ensuring that piglets had access to feed and clean drinking water ad libitum. In addition, during the first week, the ambient temperature was kept within 30–32 °C and then slowly lowered to 28–30 °C following weaning. The relative humidity was maintained between 60% and 65% throughout the experiment.

### 2.3. Growth Performance

On days 21 and 35 of lactation, individual body weights of all piglets were recorded, and residual feed from each pen was collected and weighed. From the age of 7 days to 21 days, only feed intake was measured during this period. Then, weaning weight after weaning was recorded. From the age of 21 days to 35 days, ADFI, ADG, and FBW of piglets were recorded, and the feed-to-gain ratio (F:G) was calculated. Diarrhea incidence was recorded, and the diarrhea rate was calculated according to Li et al. [13].

### 2.4. Nutrient Digestibility Assessment and Nutrient Analysis

From days 33 to 35, approximately 400 g of feces were collected from all piglets in each replicate by rectal palpation and dried at 65 °C for 72 h. Both feed and dried fecal samples were ground using a 60-mesh sieve [14]. Dry matter (DM) content, crude protein (CP), ether extract (EE), acid detergent fiber (ADF), neutral detergent fiber (NDF), and acid-insoluble ash (AIA) in diet and feces were determined according to the AOAC [15]. A Parr 6400 oxygen bomb calorimeter (Parr Instrument Company, Moline, IL, USA) was used to measure gross energy (GE). ATTD was calculated as follows:

ATTD (%) = [1 − (AIA _diet_ × Nutrient _feces_)/(AIA _feces_ × Nutrient _diet_)] × 100

### 2.5. Tissue Sample Collections

In consideration of experimental facility limitations, financial constraints, and animal welfare principles, only piglets from the meal group and 8 mm group were selected for slaughter and tissue sampling. The 8 mm group was chosen because it demonstrated the most pronounced improvements in growth performance and nutrient digestibility among all pellet treatments to investigate the underlying mechanisms of pellet diameter effects by comparing the most effective treatment with the control (meal) group. This approach, comparing only the control and best-performing extreme group, minimizes unnecessary animal use but limits the inference on dose–response relationships across pellet diameters. All mechanistic indicators (organ index, intestinal morphology, digestive enzymes, and microbiota) are, therefore, only reported for meal and 8 mm pellets, not for 2 mm or 4 mm groups.

After the experiment, a single pig with body weight nearest the average of the replicate group was selected from each replicate in the meal and 8 mm group for euthanasia and sampling. After euthanasia, intestinal segments (5 cm in length) were collected from the midpoints of the duodenum, jejunum, and ileum, rinsed with 0.9% normal saline, and immediately fixed in 10% neutral-buffered formalin for villus morphological analysis. Sterilized glass slides were used to scrape the mucosa of the duodenum, jejunum, and ileum. The samples were then placed into sterilized cryovials and stored in liquid nitrogen for the determination of maltase, lactase, and sucrase activity. The contents of the jejunum, ileum, cecum, and colon were collected, packaged in sterilized cryovials, and stored in liquid nitrogen tanks for the detection of intestinal microbiota and amylase, trypsin, chymotrypsin, and lipase activity.

### 2.6. Organ Index

After removing the residual blood from the slaughtered organs using filter paper, the organs were weighed, and the organ index was calculated using the formula presented as follows:

Organ index (g/kg) = Organ weight/Live weight before slaughter

### 2.7. Intestinal Morphology

The jejunum, ileum, and duodenal segments were fixed in 10% neutral-buffered formalin for approximately 7 days, dehydrated with alcohol, transparented with xylene, embedded in paraffin, and sliced into 5 μm sections. They were then stained with hematoxylin and eosin staining. A total of 15 typical fields of view with intact and straight villi on each tissue slice were selected, and images were collected using True Color Image Analysis software (Lucia software, Lucia, Praha, Czech Republic, version 1.2.0). VH and CD were measured. The ratio of VH to CD (V/C) was calculated, and the average of the 15 observed values was taken as statistical processing data.

### 2.8. Digestive Enzyme Activity

We accurately weighed the mucosa and chyme samples and added nine times the volume of normal saline according to the ratio of weight (g):volume (mL) = 1:9, performed mechanical homogenization under an ice-water bath, and then the homogenates were centrifuged at 2500 rpm for 10 min at 4 °C to collect the supernatant. We used kits provided by Nanjing Jiancheng Bioengineering Research Institute to detect the activities of maltase, lactase, sucrase, chymotrypsin, trypsin, amylase, and lipase through spectrophotometry. One unit (U) of enzyme activity was defined as the amount of enzyme that catalyzes the conversion of 1 μmol of substrate per minute at 37 °C. Lipase activity was expressed as ng per mg protein, consistent with the standard protocol of the diagnostic kit.

### 2.9. Microbial Communities

The extraction of total microbial genomic DNA was carried out in accordance with the E.Z.N.A. soil DNA kit (Omega Bio-tek, Norcross, GA, USA). Sequencing was performed using Illumina’s Miseq PE300 platform (Illumina, San Diego, CA, USA). Raw tags were obtained by merging paired-end reads using FLASH (version 1.2.11) software. Then, the optimized sequences were clustered into OTUs using UPARSE (version 7.1) with a 97% sequence similarity level. Following rarefaction, taxonomic annotation was conducted using the RDP classifier (version 2.11), and the taxonomic structure of the bacterial community was subsequently analyzed.

### 2.10. Statistical Analysis

The experimental unit for growth performance, nutrient digestibility, and diarrhea rate was the pen, with each treatment group consisting of six independent experimental units (*n* = 6). One piglet was selected from each replication pen for analysis of organ index and intestinal health indicators. Data from this piglet was considered representative of the pen; the experimental unit for these mechanistic parameters was the individual pig (*n* = 6 pigs per treatment).

The determination of microorganisms in the intestine was completed at Shanghai Meiji Biomedical Technology Co., Ltd. (Shanghai, China). Bioinformatic analysis of the intestinal microbiota was performed on the Majorbio Cloud platform (https://cloud.majorbio.com). Alpha diversity indices (Chao1, Shannon, etc.) were computed from OTU data using Mothur (version 1.48.3). The intestinal flora was classified at the phylum level, and the OTU richness was counted [16].

The normality of residuals and equal variances were verified using the UNIVARIATE procedure of SAS (version 9.4, Cary, NC, USA). We used the general linear model procedure of SAS to analyze data, and Tukey’s test was carried out when the differences were significant. Pellet diameter represented a quantitative factor (0, 2, 4, and 8 mm), and orthogonal polynomial contrasts were performed to determine the linear effects of pellet diameter on growth performance and nutrient digestibility using the GLM procedure in SAS. The difference in the diarrhea rate was analyzed by the chi-square test. The results were presented as mean values ± standard error of mean (SEM). A significant difference was considered at *p* < 0.05, and a trend toward significance was considered at 0.05 ≤ *p* < 0.1.

## 3. Results

### 3.1. Growth Performance

The influence of pellet diameter on the growth performance is shown in Table 3. In the first stage of the experiment, as the diameter of the feed increased, the ADFI of piglets increased significantly (*p* < 0.001) and responded linearly (*p* < 0.001) to increasing pellet diameter. In the second stage of the experiment, the FBW and ADG of piglets fed pellets were significantly higher than those of piglets fed meal feed and responded linearly (*p* < 0.05) to increasing pellet diameter, with the 8 mm group exhibiting the best growth performance (*p* < 0.05).

### 3.2. Apparent Total Tract Digestibility of Nutrients

The influence of pellet diameter on the ATTD of piglets is shown in Table 4. Overall, the ATTD of nutrients in piglets fed pellets was significantly higher than that in piglets fed meal feed and responded linearly (*p* < 0.001) to increasing pellet diameter. Moreover, as the diameter of pellets increased, the ATTD of various nutritional indicators in piglets increased significantly (*p* < 0.001), with the greatest digestibility observed in piglets fed 8 mm pellets.

### 3.3. Organ Index

The influence of pellet diameter on the organ indices is shown in Table 5. The organ index of the stomach and large intestine of piglets fed 8 mm feed was significantly lower than that of those fed meal (*p* < 0.05). No significant differences were observed in other organ indices between the two groups.

### 3.4. Intestinal Morphology

The influence of pellet diameter on the intestinal morphology of piglets is shown in Table 6. The V/C in both the jejunum and ileum of piglets fed 8 mm pellets was significantly higher than that of those fed meal feed (*p* < 0.05), and the ileal CD was significantly lower. Additionally, the jejunal VH, jejunal CD, and V/C in the duodenum showed an increasing trend (*p* < 0.1).

### 3.5. Digestive Enzyme Activity

The influence of pellet diameter on the intestinal digestive enzyme activity of piglets is shown in Table 7. The activities of amylase, trypsin, lipase, and chymotrypsin in the jejunum, as well as the activities of amylase, trypsin, and chymotrypsin in the ileum, of piglets fed 8 mm pellets were significantly higher than those fed meal feed (*p* < 0.05).

### 3.6. Microbial Communities

**Figure 1:**
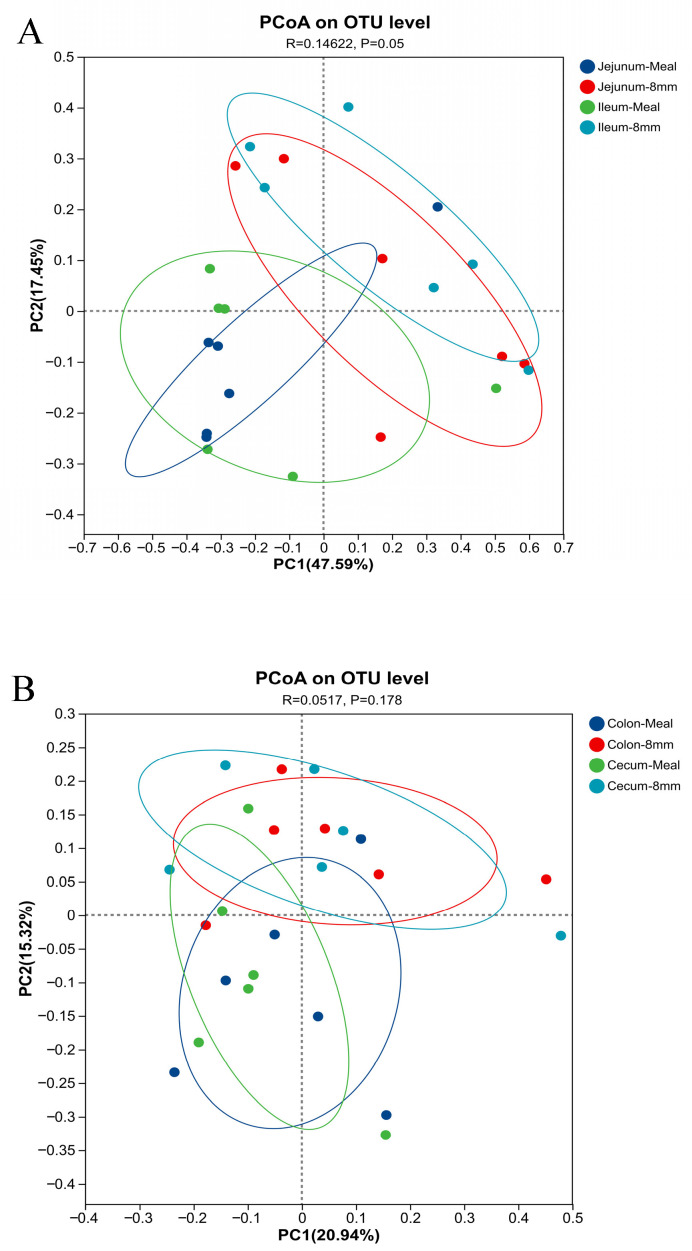
Unweighted version of the UniFrac-based PcoA for analysis of intestinal microbiota β-diversity in piglets. (**A**) PCoA analysis of β-diversity of jejunum and ileum microbiota in piglets; (**B**) PCoA analysis of β-diversity of cecum and colon microbiota in piglets. Meal = meal as the diet; 8 mm = 8 mm pellets as the diet.

**Figure 2:**
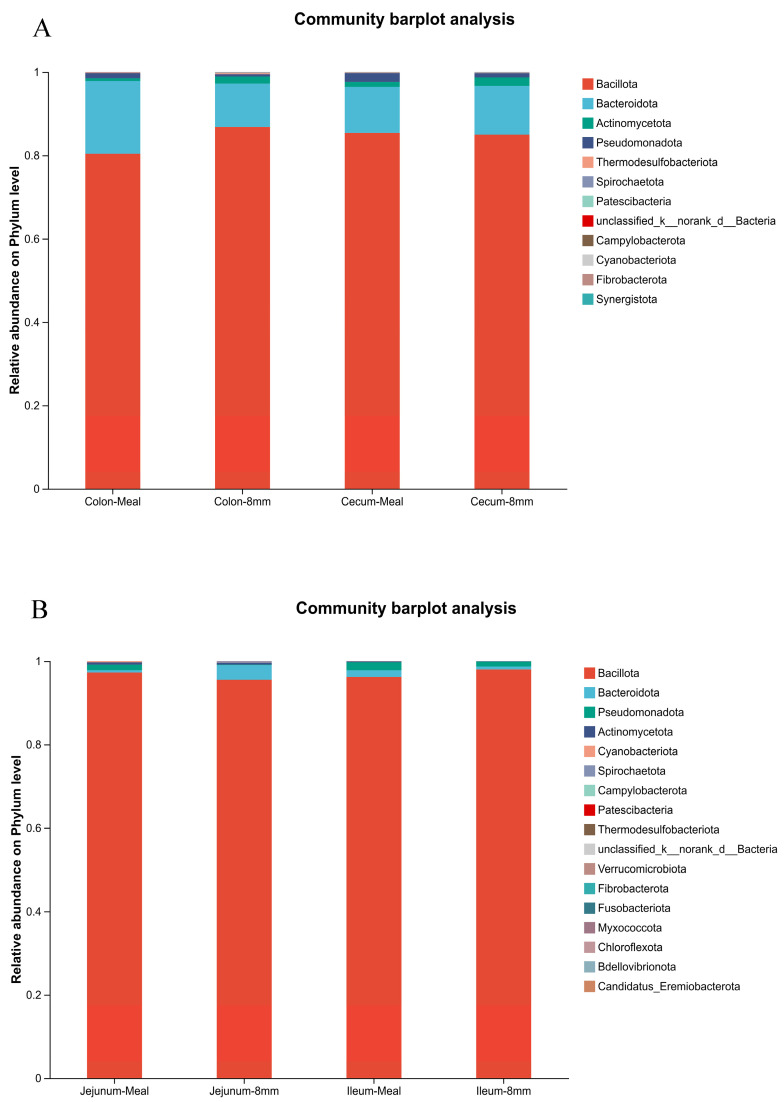
Phylum-level composition of the intestinal microbiota in piglets. (**A**) Species composition of microbiota in the jejunum and ileum of piglets at the phylum level; (**B**) species composition of microbiota in the cecum and colon of piglets at the phylum level. Meal = meal as the diet; 8 mm = 8 mm pellets as the diet.

**Figure 3:**
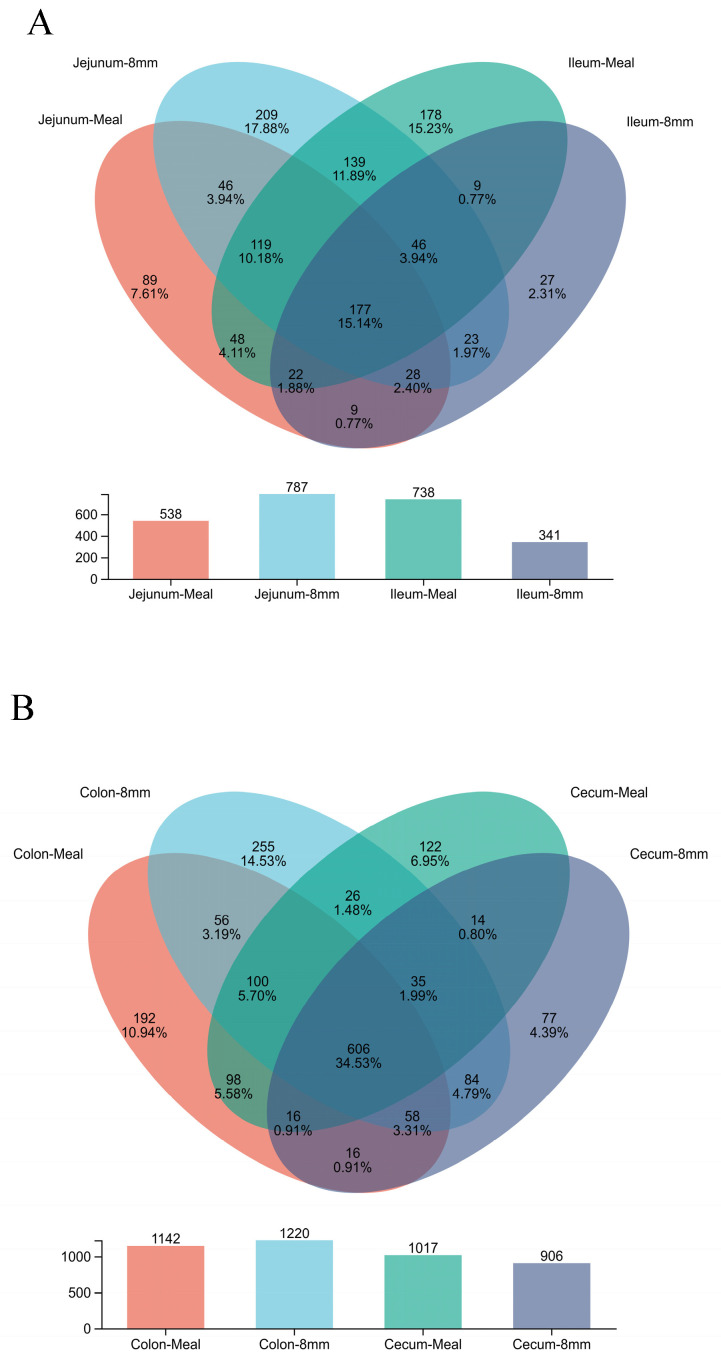
Intestinal microbial composition and diversity of suckling piglets. Note: (**A**) Microbial composition and diversity of jejunum and ileum in piglets; (**B**) microbial composition and diversity of cecum and colon in piglets. Meal = meal as the diet; 8 mm = 8 mm pellets as the diet.

In Table 8, pellet diameter did not affect alpha diversity of the intestinal microbiota in piglets. In Figure 1, PCoA showed significant differences in the bacterial community structure of the jejunum and ileum between the 8 mm group and the meal group, while the bacterial community structure of the cecum and colon did not differ. As shown in Figure 2, at the phylum level, the dominant bacterial phyla in the jejunum of piglets were Firmicutes, Pseudomonadota, and Bacteroidetes. Among them, the relative abundance of Bacteroidetes was increased, and that of Pseudomonadota was decreased in the piglets fed 8 mm pellets. The dominant bacterial phyla in the cecum and colon were Firmicutes, Pseudomonadota, and Actinobacteriota; in this regard, the relative abundance of Actinobacteriota was increased, and that of Pseudomonadota was decreased in the 8 mm group. The dominant bacterial phyla in the ileum were Firmicutes, Pseudomonadota, and Bacteroidetes, and no significant difference in the flora distribution was observed between the two treatment groups. The Venn diagram showed increased microbial richness in the jejunum and decreased microbial richness in the ileum of piglets fed 8 mm pellets, while no changes were detected in other intestinal segments (Figure 3).

## 4. Discussion

### 4.1. Growth Performance and Nutrient Digestibility

Piglets aged 7–21 days are in a critical period of transition from sow milk to solid feed, characterized by strong curiosity and high exploratory drive. Larger pellets (4–8 mm) offer greater visual and physical prominence, enhancing accessibility and exploratory feeding behavior in piglets compared with meal feed and 2 mm pellets. According to the findings of Edge et al. [17], the physical form of large pellets is more effective in stimulating the feeding instinct of piglets and increasing the frequency of their active contact with feed. The results of the present study showed that feed intake of piglets increased significantly with the increase in pellet diameter, which is consistent with previous studies [10,11,18].

Structurally, large-diameter pellets have a denser texture. In contrast, meal feed and small-diameter pellets are prone to feed wastage during ingestion and tend to disperse rapidly in the stomach with gastric juice, passing into the intestines [8]. By comparison, large-diameter pellets are less easily fragmented and enable nutrients to come into contact with digestive juices more evenly, thereby indirectly improving ATTD. The improvement of ATTD in the 8 mm group of piglets is consistent with the increase in ADG. These results suggest that large-diameter pellets significantly improved several intestinal health indicators, which is the key factor contributing to the elevated ATTD. Piglets in the 8 mm group exhibited significantly higher activities of digestive enzymes such as amylase, trypsin, and lipase in the jejunum and ileum, which may enhance nutrient digestion and absorption [19]. In addition, piglets in the 8 mm group had greater jejunal villus height, a key determinant of nutrient absorption. Longer villi increase the absorptive surface area, facilitating more efficient capture of decomposed nutrients [20]. In addition, the large-diameter pellet group exhibited an elevated proportion of beneficial intestinal bacteria and a reduced proportion of harmful bacteria. This optimized intestinal flora structure indirectly reduces nutrient loss caused by microbial competition, which may contribute to enhancing ATTD [21].

Notably, ADFI was significantly increased with a larger pellet diameter in the present study. Higher feed intake leads to greater nutrient supply, which can independently enhance apparent nutrient digestibility, stimulate digestive enzyme secretion, and support intestinal villus development and mucosal maturation. Therefore, the observed improvements in ATTD, digestive enzyme activities, and intestinal morphology in pellet groups, especially the 8 mm group, were likely driven by both the direct physical effects of pellet size and the indirect effects of increased feed and nutrient intake, rather than pellet diameter alone. These two factors interactively contribute to enhanced growth and intestinal health in creep-fed piglets.

### 4.2. Organ Index

Meal feed has a loose physical structure, and it is easy to form sticky chyme in the stomach. The contact efficiency between digestive enzymes and nutrients is low, and the stomach needs to enhance peristalsis and secrete more digestive fluid to facilitate initial digestion. Long-term feeding can cause thickening of the gastric wall muscle layer and hyperplasia of mucosal glands, which may contribute to increasing the weight of gastric organs [22]. Meanwhile, due to the lower digestibility of meal feed, undigested nutrients entering the large intestine can stimulate increased peristalsis and intestinal mucosal hyperplasia to expand the absorption area, which may contribute to compensatory weight gain in the large intestine [23] The digestion rate of 8 mm granular material is higher, and the digestion of chyme in the stomach is more complete, without the need for excessive work of the stomach and large intestine. Therefore, the organ index of the stomach and large intestine is significantly reduced in the 8 mm group than in the meal group. Furthermore, 8 mm pellets optimized intestinal morphology and enhanced digestive enzyme activity, making intestinal digestion function more mature and efficiently completing nutrient absorption without relying on increasing organ volume to compensate for functional deficiencies. Additionally, its improved gut microbiota structure can reduce intestinal inflammation and avoid organ tissue proliferation caused by inflammation [14].

### 4.3. Intestinal Morphology

VH refers to the vertical distance from the opening of the intestinal gland to the top of the villus. An increase in VH can increase the absorptive surface area of the intestinal epithelium, enhance the contact area between the small intestine and intestinal contents, and enhance digestion and nutrient absorption [24]. CD is defined as the distance from the base of the villus to the bottom of the crypt; a reduction in CD indicates improved epithelial cell maturation [25]. An increase in the ratio of the two indices signifies the enhancement of intestinal digestive and absorptive functions, enabling sufficient nutrient absorption [26]. Compared with intact pellets, meal consists of fine, loose particles that disperse rapidly in the upper gastrointestinal tract, resulting in shorter gastric retention time and less thorough digestion. As a result, nutrients are less uniformly mixed with digestive enzymes, and digesta passes through the small intestine with inconsistent contact with the mucosal epithelium. This unstable stimulation fails to promote optimal villus elongation and crypt maturation. In contrast, 8 mm pellets maintain structural integrity and larger volume, which prolongs gastric retention. The more homogeneous digesta provides sustained, gentle stimulation of the intestinal mucosa, which may contribute to greater villus height, which is consistent with the results of poultry experiments [27,28]. Additionally, since the ATTD of various nutrients in the 8 mm pellets was significantly higher than that in the meal feed group, this may have supported intestinal tissue growth, including villi development.

### 4.4. Digestive Enzyme Activity

The activity of digestive enzymes is an important indicator reflecting the intestinal health and digestive capacity of weaned piglets [29]. Large feed particles remain longer in the stomach and increase the frequency of contractions required for grinding, which may enhance mucosal stimulation and trigger the release of key digestive hormones, including cholecystokinin, gastrin, and secretin, which control pancreatic and intestinal enzyme release [27]. We speculate that the higher enzyme activities observed with 8 mm pellets were mediated by enhanced hormonal signaling triggered by improved gastric retention and mucosal sensing, consistent with classical regulatory pathways of digestion. According to our experimental results, the activity of jejunal protease, lipase, and ileal protease in piglets of the 8 mm group was significantly higher; this is consistent with the increased ATTD of CP and EE in the 8 mm group.

### 4.5. Microbial Communities

The intestinal microbiota is a complex microbial community colonizing the gastrointestinal tract, which establishes a close symbiotic relationship with the host. The results showed that dominant phyla were Firmicutes and Bacteroidetes, which were consistent with the findings of previous studies [30,31]. The higher abundance of Bacteroidetes in the 8 mm group may be attributed to increased fiber availability resulting from improved nutrient digestibility. Bacteroidetes prefer complex carbohydrates and become more abundant when simple substrates are depleted. The 8 mm pellets enhanced nutrient digestibility. More indigestible fiber flowed to the hindgut intact, while less undigested nutrients remained; thus, they became more dominant. The healthy growth of piglets relies on a dynamically balanced intestinal microecological environment, since a healthy intestinal microbiota performs functions such as promoting nutrient metabolism, maintaining the intestinal mucosal barrier, regulating immune responses, and inhibiting pathogen infection [32].

Large-diameter pellets have higher ATTD, resulting in less undigested nutrients entering the posterior intestinal tract, with the residues mainly being indigestible crude fiber. Some Pseudomonadota, such as *Pseudomonas aeruginosa*, are pathogenic and harmful, and they can cause animal infections and hinder growth and development. These bacteria are highly dependent on simple carbon sources, such as glucose, for rapid proliferation [33,34]. However, such nutrients are scarce in the 8 mm group, which inhibits the growth of harmful bacteria due to nutrient deficiency. On the contrary, Bacteroidetes in the intestine, as common beneficial bacteria, play important roles in immune regulation, intestinal barrier integrity, and colonization resistance. They are adept at decomposing complex carbohydrates and can utilize residual crude fiber as a nutrient source for proliferation [35,36], ultimately leading to an increased proportion. In addition, the results of the present study indicate that large-diameter pellets can increase intestinal VH, reduce CD, and enhance intestinal barrier function. A healthy intestinal mucosa can secrete mucin to protect the intestinal wall and provide adhesion sites for beneficial bacteria, facilitating their stable growth in the intestine [31]. At the same time, the strong barrier function can prevent harmful bacteria from penetrating the intestinal wall, reduce the damage of harmful bacteria to the host, and indirectly inhibit the growth of harmful bacteria. In contrast, meal feed has lower digestibility, and more undigested simple sugars and proteins enter the distal intestine tract, resulting in rapid reproduction and an increased proportion of harmful bacteria. Meanwhile, excessive nutrient residues will also intensify nutrient competition among microbiota, which may reduce the ecological niche of beneficial bacteria and cause an imbalance in the microbiota structure.

The jejunum, as the core intestinal segment for nutrient absorption, still retains some incompletely digested nutrients, which provide abundant carbon and nitrogen sources for microorganisms. Meal feed has lower digestibility; some nutrients may be excessively consumed or wasted in the stomach and duodenum, resulting in insufficient available nutrients in the jejunum and thus limiting microbial growth. Meanwhile, 8 mm pellets have a higher digestibility and improve the integrity of the intestinal mucosal barrier. The healthy mucosal surface can secrete substances such as mucin, providing stable adhesion sites for microorganisms [31] and reducing the loss of microorganisms with chyme, which may contribute to increasing the microbial relative richness in the jejunum.

The ileum is located after the jejunum and serves as the final segment for nutrient absorption [37]. The 8 mm pellets have higher digestibility in the jejunum; most available nutrients are fully absorbed, leading to a significant reduction in residual nutrients entering the ileum, which are mainly composed of indigestible crude fiber and complex polysaccharides. We speculate that this nutrient-deficient environment exerts a selective effect on microorganisms, resulting in a decrease in the microbial OTU richness of the ileum. In contrast, meal has low digestibility in the jejunum; a large amount of undigested simple nutrients enter the ileum, providing survival conditions for various microorganisms, which may contribute to the higher OTU richness.

The cecum and colon are the main colonization regions of intestinal microbiota, and their nutrient sources are mainly composed of undigested crude fiber and dietary fiber from the anterior intestinal tract [38]. For both pellets and meal feed, the nutrients entering the cecum and colon are dominated by indigestible components. Moreover, the microbial community in the cecum and colon is complex, stable, and functionally redundant [38], resulting in no differences in microbial diversity between treatments.

### 4.6. Limitations

This study has a key limitation. Mechanistic data (intestinal morphology, digestive enzyme activities, and gut microbiota) were only measured in the meal and 8 mm pellet groups (the two extreme treatments), while the 2 mm and 4 mm pellet groups were not sampled for mechanistic analysis. Therefore, although growth performance and nutrient digestibility showed a numerical dose–response pattern with increasing pellet diameter, no dose–response inference can be made for intestinal health and mechanistic endpoints. Conclusions regarding intestinal morphology, enzyme activities, and microbiota are only valid for meal vs. 8 mm pellets and cannot be directly extrapolated to intermediate pellet diameters (2 mm and 4 mm).

Another limitation is the lack of direct gut health biomarker measurements, such as intestinal permeability, inflammatory cytokines, tight junction protein expression, or oxidative stress indicators. Although intestinal morphology, digestive enzyme activities, and microbial community structure are widely used as important indicators of intestinal health status, direct molecular or physiological biomarkers of gut barrier function and inflammatory status were not determined in this experiment.

## 5. Conclusions

In summary, 8 mm pellets significantly improved the growth performance of piglets, as indicated by greater FBW and ADG post-weaning and increased ATTD of nutrients. Additionally, 8 mm pellets showed the greatest numerical improvement and were the only size to consistently outperform the meal feed across all growth metrics. Compared with meal, 8 mm pellets also optimized intestinal morphology by increasing the height of jejunal villi and reducing the depth of ileal crypts, increasing digestive enzyme activities in the jejunum and ileum, and improving intestinal microbiota composition by increasing the abundance of beneficial bacteria. Overall, 8 mm pellets improved the growth performance, enzyme activity, and microbial composition in piglets. From a practical perspective, they are feasible and practical for commercial feed production.

## Figures and Tables

**Table 1:** Composition and calculated nutrients of basal diets (as-fed basis, %).

Items	Contents %
Ingredients	
Corn	31.50
Extruded corn	20.90
Soybean meal	12.00
Fermented soybean meal	5.00
Fish meal	2.00
Whey meal	7.00
Extruded soybean	10.00
Dicalcium phosphate	1.50
Limestone	0.75
Salt	0.30
Soybean oil	2.00
Soybean protein concentrate	2.00
Glucose	2.00
White sugar	1.00
Lysine	0.50
Methionine	0.20
Threonine	0.20
Tryptophan	0.05
Premix ^1^	0.50
Choline	0.10
Acidifier	0.50
Total	100.00
Calculated nutrients ^2^	
Gross energy (MJ/kg)	14.36
Crude protein	18.98
Calcium	0.81
Phosphorus	0.65
Lysine	1.51
Methionine	0.49
Threonine	0.91
Tryptophan	0.25

^1^ The premix supplied the following nutrients per kg of diet: vitamin A, 12,000 IU; vitamin E, 24 IU; vitamin K3, 2.0 mg; vitamin B6, 3.0 mg; vitamin B12, 24 μg; vitamin B2, 6.0 mg; vitamin B1, 2.0 mg; nicotinic acid, 30 mg; D-pantothenic acid, 20 mg; folic acid, 3.6 mg; biotin, 0.1 mg; choline chloride, 0.4 mg; Cu, 8.0 mg as CuSO_4_·5H_2_O; Fe, 96 mg as FeSO_4_·H_2_O; Zn, 120 mg as ZnSO_4_·H_2_O; Mn, 40 mg as MnSO_4_·H_2_O; I, 0.56 mg as Ca(IO_3_)_2_; Se, 0.4 mg as sodium selenite. ^2^ Nutrient levels were estimated based on the NRC [12].

**Table 2:** Physical quality of three types of pellets ^1^.

Items ^2^	Treatments ^3^			SEM ^4^	*p*-Value
	2 mm	4 mm	8 mm		
PDI (%)	98.68 ^a^	96.60 ^a^	96.33 ^b^	0.472	0.001
Hardness (kg)	43.83 ^a^	54.67 ^b^	66.01 ^b^	41.66	<0.001

^1^ In the same row, values with different superscript lowercase letters indicate differences (*p* < 0.05). ^2^ PDI = pellet durability index. ^3^ 2 mm = 2 mm pellets as the diet; 4 mm = 4 mm pellets as the diet; 8 mm = 8 mm pellets as the diet. ^4^ SEM = standard error of mean (*n* = 10).

**Table 3:** The influence of pellet diameter on the growth performance of piglets ^1^.

Items ^2^	Treatments ^3^				SEM ^4^	*p*-Value	*p*-Linear
	Meal	2 mm	4 mm	8 mm			
7–21 day							
FBW (kg)	7.40	7.90	8.05	7.72	0.126	0.298	0.112
ADFI (g)	12.50 ^d^	35.71 ^b^	25.00 ^c^	48.95 ^a^	5.458	<0.001	<0.001
21–35 day							
FBW (kg)	8.70 ^b^	9.57 ^ab^	9.53 ^ab^	10.19 ^a^	0.180	0.031	0.018
ADFI (g)	161.85	203.29	167.60	240.48	17.071	0.383	0.165
ADG (g)	144.18 ^b^	163.16 ^ab^	147.61 ^b^	190.67 ^a^	5.424	0.006	0.003
F:G	1.14	1.36	1.17	1.36	0.072	0.623	0.310
Diarrhea rate (%)	1.39	1.79	1.79	2.78	0.162	0.290	0.145

^1^ In the same row, values with different superscript lowercase letters indicate differences (*p* < 0.05). ^2^ FBW = final body weight; ADFI = average daily feed intake; ADG = average daily gain; F:G = the ratio of feed to gain. ^3^ Meal = meal as the diet; 2 mm = 2 mm pellets as the diet; 4 mm = 4 mm pellets as the diet; 8 mm = 8 mm pellets as the diet. ^4^ SEM = standard error of mean (*n* = 6).

**Table 4:** The influence of pellet diameter on the ATTD of piglets ^1^.

Items ^2^	Treatments ^3^				SEM ^4^	*p*-Value	*p*-Linear
	Meal	2 mm	4 mm	8 mm			
Dry matter (%)	82.93 ^d^	85.42 ^c^	86.40 ^b^	87.37 ^a^	0.358	<0.001	<0.001
Ash (%)	50.86 ^c^	52.49 ^c^	58.61 ^b^	60.84 ^a^	0.697	<0.001	<0.001
EE (%)	63.83 ^c^	70.95 ^b^	74.54 ^b^	80.07 ^a^	0.663	<0.001	<0.001
CP (%)	78.56 ^c^	81.91 ^b^	83.25 ^ab^	84.76 ^a^	0.470	<0.001	<0.001
NDF (%)	55.75 ^c^	60.41 ^b^	65.04 ^a^	67.44 ^a^	1.018	<0.001	<0.001
ADF (%)	47.35 ^d^	56.02 ^c^	62.47 ^b^	66.63 ^a^	1.423	<0.001	<0.001
GE (%)	83.20 ^d^	86.07 ^c^	87.07 ^b^	87.81 ^a^	0.367	<0.001	<0.001

^1^ In the same row, values with different superscript lowercase letters indicate differences (*p* < 0.05).^2^ EE = ether extract; CP = crude protein; NDF = neutral detergent fiber; ADF = acid detergent fiber; GE = gross energy.^3^ Meal = meal as the diet; 2 mm = 2 mm pellets as the diet; 4 mm = 4 mm pellets as the diet; 8 mm = 8 mm pellets as the diet. ^4^ SEM = standard error of mean (*n* = 6).

**Table 5:** The influence of pellet diameter on the organ indices of piglets ^1^.

Items	Treatments ^2^		SEM ^3^	*p*-Value
	Meal	8 mm		
Stomach (g/kg)	10.25 ^a^	7.97 ^b^	0.781	0.015
Liver (g/kg)	35.15	33.49	3.440	0.640
Spleen (g/kg)	2.14	2.27	0.337	0.703
Pancreata (g/kg)	2.95	2.58	0.243	0.154
Small intestine (g/kg)	52.43	52.86	4.915	0.932
Large intestine (g/kg)	27.53 ^a^	20.51 ^b^	2.673	0.034

^1^ In the same row, values with different superscript lowercase letters indicate differences (*p* < 0.05). ^2^ Meal = meal as the diet; 8 mm = 8 mm pellets as the diet. ^3^ SEM = standard error of mean (*n* = 6).

**Table 6:** The influence of pellet diameter on the intestinal morphology of piglets ^1^.

Items ^2^	Treatments ^3^		SEM ^4^	*p*-Value
	Meal	8 mm		
VH (μm)				
Ileum	455.18 ^b^	701.15 ^a^	74.096	0.008
Jejunum	477.46	626.77	72.401	0.066
Duodenum	600.60	697.36	90.255	0.309
CD (μm)				
Ileum	349.95 ^b^	250.49 ^a^	26.873	0.004
Jejunum	431.54	277.78	73.257	0.085
Duodenum	501.73	439.25	37.745	0.129
V/C				
Ileum	1.30	2.83	0.244	<0.001
Jejunum	1.16 ^b^	2.30 ^a^	0.250	0.004
Duodenum	1.21	1.61	0.197	0.072

^1^ In the same row, values with different superscript lowercase letters indicate differences (*p* < 0.05). ^2^ VH = villus height; CD = crypt depth; V/C = the ratio of villus height to crypt depth.^3^ Meal = meal diet; 8 mm = pellet diet with diameter of 8 mm. ^4^ SEM = standard error of mean (*n* = 6).

**Table 7:** The influence of pellet diameter on the intestinal digestive enzyme activity of piglets ^1^.

Items ^3^	Treatments ^2^		SEM ^4^	*p*-Value
	Meal	8 mm		
Jejunum				
Maltase (U/mg)	89.49	70.37	11.477	0.127
Lactase (U/mg)	20.97	11.93	4.479	0.102
Sucrase (U/mg)	47.33	29.22	10.155	0.130
Amylase (U/mg)	0.20 ^b^	0.34 ^a^	55.565	0.030
Trypsin (U/mg)	0.09 ^b^	0.15 ^a^	23.172	0.031
Lipase (ng/mg)	160.36 ^b^	215.12 ^a^	16.455	0.008
Chymotrypsin (U/mg)	0.17 ^b^	0.24 ^a^	27.528	0.047
Ileum				
Maltase (U/mg)	56.25	49.11	8.855	0.439
Lactase (U/mg)	5.11	5.57	0.707	0.535
Sucrase (U/mg)	14.29	18.78	2.935	0.158
Amylase (U/mg)	0.14 ^b^	0.27 ^a^	35.035	0.004
Trypsin (U/mg)	0.08 ^b^	0.13 ^a^	12.216	0.002
Lipase (ng/mg)	110.76	134.09	12.370	0.089
Chymotrypsin (U/mg)	0.13 ^b^	0.19 ^a^	19.150	0.013
Duodenum				
Maltase (U/mg)	39.93	32.50	5.476	0.204
Lactase (U/mg)	4.01	4.55	0.894	0.565
Sucrase (U/mg)	8.43	8.44	2.058	0.995

^1^ In the same row, values with different superscript lowercase letters indicate differences (*p* < 0.05). ^2^ Meal = meal as the diet; 8 mm = 8 mm pellets as the diet. ^3^ One unit (U) of enzyme activity was defined as the amount of enzyme that catalyzes the conversion of 1 μmol of substrate per minute at 37 °C. ^4^ SEM = standard error of mean (*n* = 6).

**Table 8:** The influence of pellet diameter on the alpha diversity of intestinal microbiota in piglets.

Items	Treatments ^1^		SEM ^2^	*p*-Value
	Meal	8 mm		
Cecum				
Ace	489.90	458.83	32.493	0.655
Chao	481.62	450.22	31.560	0.642
Shannon	3.57	3.60	0.112	0.893
Simpson	0.073	0.069	0.008	0.828
Sobs	411.50	386.33	30.151	0.697
Colon				
Ace	584.43	604.09	34.152	0.789
Chao	569.66	583.72	32.958	0.843
Shannon	3.76	3.92	0.134	0.577
Simpson	0.066	0.053	0.010	0.510
Sobs	492.83	521.00	33.120	0.691
Ileum				
Ace	254.64	176.98	50.063	0.464
Chao	240.03	150.13	48.332	0.377
Shannon	2.01	1.28	0.233	0.120
Simpson	0.28	0.45	0.055	0.122
Sobs	199.67	111.33	42.609	0.341
Jejunum				
Ace	323.88	301.91	56.188	0.856
Chao	294.48	290.85	55.341	0.976
Shannon	1.85	1.77	0.295	0.899
Simpson	0.26	0.41	0.057	0.183
Sobs	204.83	230.33	48.276	0.806

^1^ Meal = meal as the diet; 8 mm = 8 mm pellets as the diet. ^2^ SEM = standard error of mean (*n* = 6).

## Data Availability

Data can be provided by the corresponding author upon reasonable request.
